# Books Reviewed

**Published:** 1879-06

**Authors:** 


### Books Reviewed.

*A Practical Treatise on the Diseases of the Testis and of the Spermatic Cord and Scrotum.* By T. B. Curling, F. R. S., Consulting Surgeon to the London Hospital. Fourth Edition, Revised and enlarged. Philadelphia: Lindsay & Blakiston. T. H. Butler, Buffalo.

This book opens up to us a field for a new specialty not yet *advertised* by the regular profession, viz: *Diseases of Males.* This will yet offset the gyneacological section of special workers in the Diseases of Females. After reading this work, we are strongly impressed with the idea that here is now open a most promising field for special workers, though most of the topics have heretofore been included in comprehensive works upon general surgery. Our author sustains his positions by most painstaking reports of illustrative cases, and his views are unmistakably orthodox upon the various subjects which he discusses. Diseases of the testis are treated with a completeness which leaves nothing to be added; this will be appreciated when we say that this comprises 519 pages of the work, while the remainder of the book is devoted to diseases of the spermatic cord and to diseases of the scrotum.

---

*An Introduction to Pathology and Morbid Anatomy.* By T. Henry Green, M. D., London, Fellow of the Royal College of Physicians, London; Physician to Charing Cross Hospital, and Lecturer on Pathology and Morbid Anatomy at Charing Cross Hospital Medical School; Assistant Physician to the Hospital for Consumption and diseases of the Chest. Brompton, Third American from the fouth revised and enlarged English edition, with one hundred and thirty-two illustrations. Philadelphia: Henry C. Lea, 1878. T. H. Butler, Buffalo.

Our author gives us concise, complete, and beautifully illustrated chapters upon the most interesting of all subjects connected with the science of medicine. The real student of health and disease, will find the foundation of knowledge in disease embodied in the pages of this volume. The first chapter upon the Cell as seat of Nutrition and Function, Constitution of Cells, Protoplasm, Nucleus, Cell-Wall, Physiology of Cells, Genesis of Cells, Vacuolation &c., &c., attracts our attention as worthy of special mention, its illustrative cuts so symplifying its teaching as to be understood by early students in pathology, and at once lending attraction to an otherwise somewhat intricate study.

---

*Conspectus of Organic Materia Medica and Pharmacal Botany,* Comprising the Vegetable and Animal Drugs; their physical character, geographical origin, classification, constituents, doses, adulterations &c., table of the tests and solubilities of the alkaloids appended. By L. E. Sayre, P. H. G. Philadelphia: D. G. Brinton, 115 South Seventh street, 1879.

A chart begins the book which gives the medicinal properties, doses &c., so arranged that it can be readily referred to and studied.

The subjects of Medical Botany and Materia Medica are divided in systematic manner, and illustrated so as to make them attractive and instructive to every attentive student of materia medica.

A complete table of the Alkaloids is presented, giving their tests and solubilities &c., also a table of antidotes and incompatibles. As a whole it may be called a valuable book of ready reference.

*A Manual of the Diseases of Children, with a Formulary.* By EDWARD ELLIS, M. D., late Senior Physician to the Victoria Hospital for sick children. Woods Library of Standard Medical Authors.

The definite, plain and practical teaching of the author is every where apparent, making it an infallible guide in the treatment of diseases of children. The author speaks from personal, independant knowledge, as one having authority.

One remarkable feature of the book is, that so complete and comprehen_ sive a work can be furnished by Wm. Wood & Co., at so small a price; a whole Library of Practical Medicine for twelve dollars.

---

*Differential Diagnosis.* A manual of the comparative Gemeiology of the more important Diseases. By F. DE HAVILLAND, M. D., Assistant Physician to the Westminster Hospital, London. Philadelphia: D. G. Brinton, 115 South Seventh street.

This book presents in tabular form the comparative points of diagnosis in many important diseases, and to the young practitioner and student is a ready helper in diagnosis. It presents in a comprehensive and clear manner the chief distinctions which are to be observed in diseases which resemble, or are liable to be mistaken for each other, and to the young or inexperienced must be regarded as a valuable guide.

---

*Modern Medical Therapeutics*; A compendium of recent Formulæ and specific Therapeutical Directions, from the practice of eminent contemporary physicians, American and Foreign. By GEORGE H. NAPHEYS, A. M., M. D., etc. Sixth edition enlarged and revised. Philadelphia: D. G. Brinton, 115 South Seventh street, 1879.

The early appearance of this Sixth Edition of Naphey's Medical Therapeutics was to be expected, and shows with what universal favor and approval the work has been reeeived by the Profession. We have little to add to our review of recent date, other than to say, that it has been carefully revised, and important recent therapeutic knowledge added to the work. It is of universally acknowledged value and too much cannot be said in its favor; space only prevents our pointing out again its points of chief value. Naphey's Medical Therapeutics when joined with a work by the same author upon Surgical Therapeutics, constitute an almost complete Library of practical Medicine, and should be in the hands of every ambitious progressive physician.

